# New species of smiley-faced spider *Spintharus* (Araneae, Theridiidae) from Brazil, and comments on unobserved diversity in South America

**DOI:** 10.3897/zookeys.915.47563

**Published:** 2020-02-24

**Authors:** Gabriel A. LeMay, Ingi Agnarsson

**Affiliations:** 1 Department of Biology, University of Vermont, 109 Carrigan Drive, Burlington, VT, 054050086, USA University of Vermont Burlington United States of America; 2 Department of Entomology, National Museum of Natural History, Smithsonian Institution, 10th & Constitution NW Washington, DC 20560-0105, USA Smithsonian Institution Washington United States of America

**Keywords:** Biogeography, cobweb spiders, cryptic species, DNA barcoding

## Abstract

*Spintharus* is a genus of spiders that contained only two species until 2018 when it was demonstrated that a ‘widespread’ species was instead composed of multiple short-range endemics. This note redescribes *Spintharus gracilis* Keyserling and describes a new species of *Spintharus* (Araneae, Theridiidae), *S. leverger***sp. nov.**, both based on specimens from Brazil. We also examine specimens from several additional localities in Brazil displaying variation consistent with patterns previously found within the Caribbean: geographically isolated and unique localities may contain independent species lineages. Given the limited number of specimens, profuse variation, and lack of DNA data from museum specimens, it is challenging to gauge the number of species in the observed material. Instead of describing these as new species here, we highlight this variation and hypothesize that in South America, a greater diversity of the genus across the geographical landscape will be found than predicted based on Levi’s “widespread *Spintharus flavidus*” hypothesis. Our results suggest that continental efforts to sample the genus would be profitable, as this charismatic group likely harbors unappreciated diversity throughout the continent.

## Introduction

American cobweb spiders (Theridiidae) were revised by Herbert Levi in numerous taxonomical treatises throughout the 1950s and 1960s (e.g. Levi 1954, 1955, 1959, 1963a, b). Many of the genera, including *Spintharus*, were subsequently placed in morphological (Agnarsson 2004) and molecular phylogenetic analyses (Arnedo et al. 2004, Liu et al. 2016), largely supporting Levi’s work. In his deliberate and practical treatment of all American araneids, tetragnathids, and theridiids Levi addressed the genus *Spintharus* in two papers (Levi 1954, 1963a). Therein he redescribed *S. gracilis* Keyserling from Brazil, and concluded that only one other species could be recognized: *S. flavidus* Hentz that he characterized as an “extremely variable” and widespread species (Levi 1954: 81). As indicated by later molecular work and systematic revisions (Dziki et al. 2015, Agnarsson et al. 2018), the profuse variation described by Levi appears to represent a large species complex, rather than one or a few highly variable species. However, the morphological variation makes organization into species level bins challenging, and some related species are arduous to diagnose based on morphology alone. Hence, Agnarsson et al. (2018) relied heavily on molecular diagnoses, utilizing DNA barcodes.

To date, only taxa from the Caribbean region have been revisited since Levi’s revisions, but here we examine samples made available by loan from Brazil provided by the Museu de Ciências e Tecnologia Pontifícia (MCTP), Rio Grande do Sul. We find clear morphological differences between some of the specimens found at different locations, suggesting that the pattern found throughout the Caribbean of diverse, potentially speciose groups remaining undescribed will likely hold on the South American continent as well. Here, we redescribe *S. gracilis*, and describe a new species in order to call attention to likely overlooked diversity and the need for extensive sampling of fresh DNA grade material of the genus across South and Central America.

## Material and methods

Specimens were deposited in the following collection: MCTP, Coleção de Aracnídeos, Porto Alegre (curator: Renato Augusto Teixeira) and the Smithsonian Museum of Natural History (SMNH), Arachnida and Myriapoda collection, Washington DC (curator: Hannah Wood). We attempted DNA extraction and amplification of DNA barcodes from legs of borrowed specimens preserved in ethanol, however this yielded no viable DNA. Specimens were then mounted for photography by being partially submerged in a Petri dish of ethyl alcohol gel (hand sanitizer) for stabilization, and then fully covered with liquid ethyl alcohol to prevent glare. Photographs were taken in the dorsal, ventral, and lateral views of the specimen with a Canon 5D camera with a 65mm macro zoom lens. Multiple photographs taken with a narrow depth of field were combined using Helicon Focus software to present a single photograph of the entire specimen in detail. Measurements were performed in Adobe Photoshop using camera lens and zoom magnification to determine scale. Epigyna were photographed before being dissected from the specimen. Soft tissue was then dissolved using a diluted potassium hydroxide solution and cleared epigyna were photographed in ventral and dorsal views. Left male pedipalps were removed before being photographed. Diagnostic descriptions of abdominal morphological features follow (Agnarsson et al. 2018).

## Taxonomy

### Spintharus gracilis

Taxon classificationAnimaliaAraneaeTheridiidae

Keyserling, 1886

49EE210D-B312-5118-8C60-F50BA272ABA1

Figure 1A–O

Spintharus gracilis Keyserling, 1886: 244, plate 20, fig. 298a, b (Holotype unknown, however syntypes from Blumenau, Santa Catarina, Brazil, deposited in the British Museum of Natural History have been re-examined; Levi 1963a: 227, figs 2v, 10–13).

#### Material examined.

**Brazil**, Rio Grande do Sul, São Leopoldo, 19.viii.1986, C.J. Becker, 1 female, (MCTP); São Leopoldo, 19.viii.1986, C.J. Becker, 1 male, (MCTP); Eldorado do Sul, 30°05'32.2"S, 51°40'20.4"W, 25.vii.1995, A.A. Lise, 1 male, (MCTP); Novo Hamburgo, 1.x.1986, C.J. Becker, 2 females, 1 male, (MCTP); Campo Bom, 19.x.1987, C.J. Becker, 1 male, (MCTP); Viamão, 30°04'42.7"S, 51°03'02.0"W, 19.viii.1994, A.A. Lise, 1 female, 4 males, (MCTP).

#### Diagnosis.

**Figure 1.**
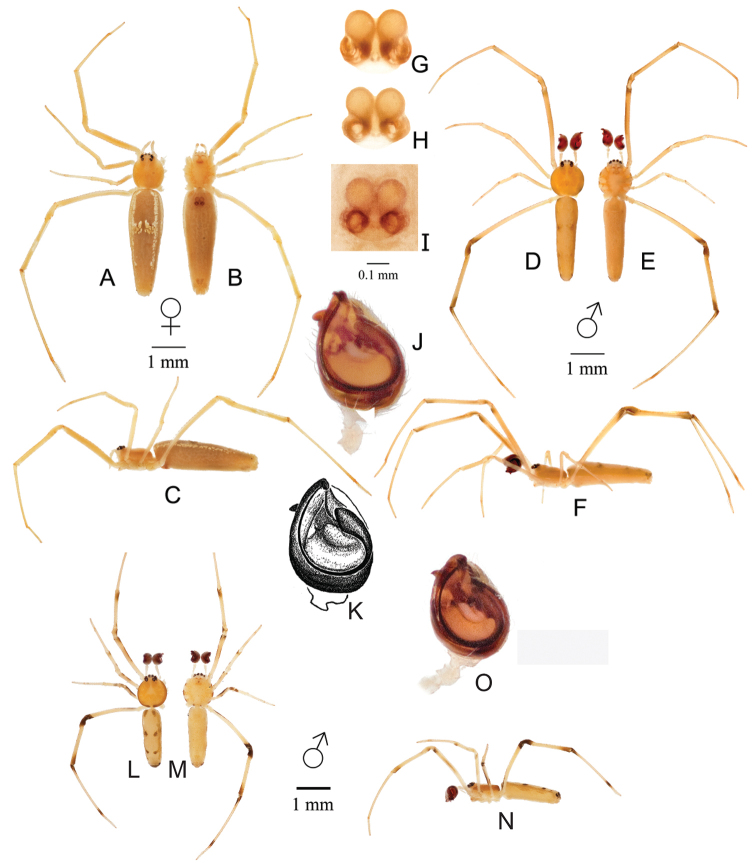
*Spintharus gracilis* Keyserling from Rio Grande do Sul, São Leopoldo. Female (**A–C**); **A** dorsal **B** ventral **C** lateral. Male (**D–F**); **D** dorsal **E** ventral **F** lateral **G–I** epigynum: **G** digested dorsal **H** digested ventral **I** undigested ventral **J** palp ventral **K** male syntype palp illustrated by Levi (1963a). **L–N** Male from Rio Grande do Sul, Eldorado do Sul (30°05'32.2"S, 51°40'20.4"W) **L** dorsal **M** ventral **N** lateral **O** palp ventral. While we hypothesize that all illustrated palps belong to *S. gracilis*, note that the male from Eldorado do Sul is smaller, has smaller palp, and differs subtly in conformation, e.g., area of tegulum exposed.

*Spintharus gracilis* females differ from all other *Spintharus* species by the long and narrow abdomen being >3× longer than wide (Fig. 1A–C). Males differ from all other *Spintharus* species by the extremely long embolus traversing the entire outer edge of the tegulum (Fig. 1J, K, O).

#### Description.

**Female.** Total length 4.21 (mm). Cephalothorax 1.05 long, 0.92 wide, 0.67 high, light yellow.

Sternum 0.77 long, 0.51 wide, extending half way between coxae IV, light yellow. Abdomen 3.16 long, 1.04 wide, 0.88 high. Narrow to oval without humps (Fig. 1A–C). Fragmented white lines follow the dorsolateral edge from anterior to posterior, excluding the posterior third of the total abdomen length. Terminuses of white lines are inflected slightly medially, being more pronounced at the anterior terminus. White markings nearly join to form a strip just anterior of the abdomen center, but remain separated by an unpigmented gap. All eyes approximately equal in size, anterior median eyes 0.06 in diameter, anterior lateral eyes 0.11 in diameter. All eyes slightly elevated on cephalothorax and located within one eye diameter apart from each other, except the posterior median, which are 0.18 apart. Leg I femur 1.92, patella 0.45, tibia 1.37, metatarsus 1.90, tarsus 0.59. All legs pale yellow.

Epigynum with widely spaced and distinctly round copulatory openings and copulatory ducts spirals extending beyond the ectal margin of spermathecae.

**Male.** Total length 3.56. Cephalothorax 0.97 long, 0.99 wide, 0.60 high, yellow with slightly darker shading on lateral sides. Sternum 0.69 long, 0.50 wide, extending half way between coxae IV, light yellow. Abdomen 2.5 long, 0.67 wide, 0.71 high. All eyes approximately equal in size, anterior median eyes 0.09 in diameter, anterior lateral eyes 0.12 in diameter. All eyes slightly elevated on cephalothorax and located within one eye diameter apart from each other, except the posterior medians, which are 0.18 apart. Leg I femur 2.28, patella 0.41, tibia 1.58, metatarsus 2.12, tarsus 0.50. All legs yellow. Darker brown shading on leg IV on patella and where tibia meets metatarsus.

Male pedipalp with an extremely long spiral traversing the entire outer edge of the tegulum, leaving a large area of the tegulum exposed (Fig. 1J).

**Taxonomic note.** The specimens examined here are from the southeast coast of Brazil but to the south of the hitherto documented locations. Given the strong genetic structure found in the Caribbean (Dziki et al. 2015) over relatively short distances, we cannot rule out that our redescription represents a new species. However, detailed sampling coupled with DNA data will be necessary to test the limits of *S. gracilis*, as was the case for “*S. flavidus*” (Agnarsson et al. 2018).

### Spintharus leverger sp. nov.

Taxon classificationAnimaliaAraneaeTheridiidae

C5D53D6B-3C42-5479-9DDC-E54F0EA5EE36

http://zoobank.org/BCA7D823-032D-417B-973B-9C2F5D9CF376

Figure 2A–N

#### Type material.

***Holotype*** female from Santo Antônio de Leverger, Mato Grosso, **Brazil**, 29.vii.1992, A.A. Lise & A. Braul, in MCTP. ***Paratype*** male with same data, deposited in the SMNH.

#### Additional material.

**Brazil**: Mato Grosso, Santo Antônio de Leverger, 29.vii.1992, A.A. Lise & A. Braul, 4 females, 1 male, (MCTP); Chapada dos Guimarães, 15–26.vii.1992, A.A. Lise & A. Braul, 1 male, (MCTP); Rio Grande do Sul, Quaraí, 4–28.v.1991, A.A. Lise, 2 females, (MCTP)

#### Diagnosis.

**Figure 2.**
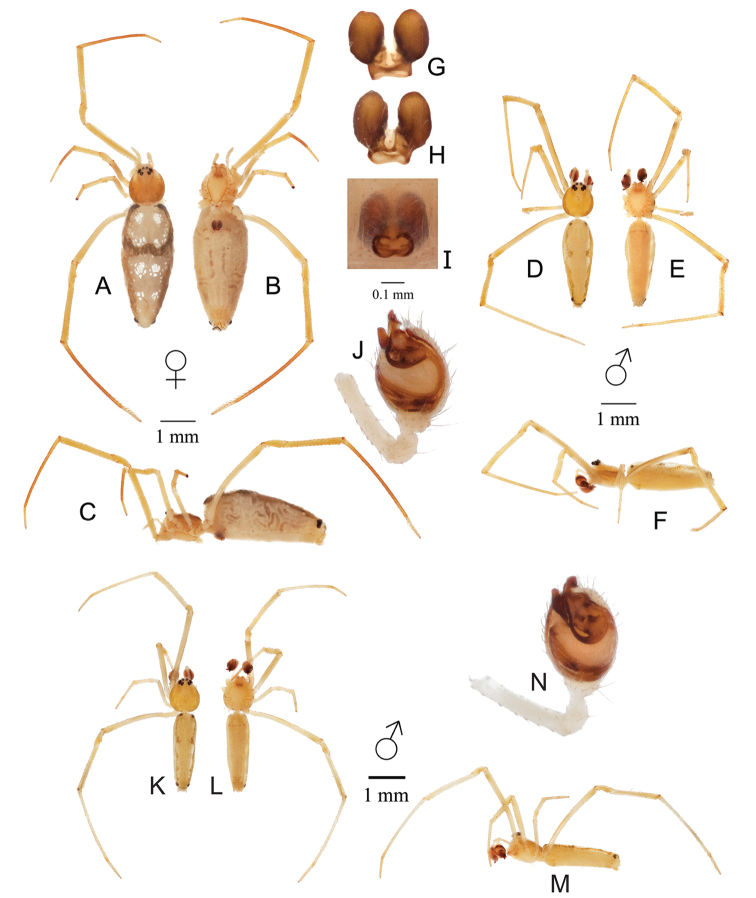
*Spintharus leverger* LeMay & Agnarsson, sp. nov. Holotype female and paratype male from Mato Grosso, Santo Antônio de Leverger. Female (**A–C**); **A** dorsal **B** ventral **C** lateral. Male (**D–F**); **D** dorsal **E** ventral **F** lateral **G–I** epigynum **G** digested dorsal **H** digested ventral **I** undigested ventral **J** palp ventral. Male from Mato Grasso, Chapada dos Guimarães. Male (**K–M**); **K** dorsal **L** ventral **M** lateral **N** male pedipalp, ventral. Note that male from Chapada dos Guimarães has sclerites slightly less juxtaposed at the terminus of the palp.

*Spintharus leverger* sp. nov. differ from all other *Spintharus* species by the large and robustly sclerotized spermathecae of the female genitalia (Fig. 2G–I) and the tight distal compression of male palpal sclerites, with most of the tegulum exposed (Fig. 2N).

#### Description.

**Female**: Total length 4.72. Cephalothorax 1.10 long, 1.03 wide, 0.82 high, yellow-brown with slightly darker shading laterally. Sternum 0.90 long, 0.58 wide, extending half way between coxae IV, yellow. Abdomen 3.50 long, 1.53 wide, 1.50 high, elongated oval without humps (Fig. 2A–C). Four pairs of juxtaposed clustered white blotches run along the dorsal side. White blotches appear slightly fragmented. Brown markings follow the dorsolateral edges of the abdomen from anterior to posterior, joining together just anterior of the center of the abdomen to form a narrow strip. A pair of small dark bulbous spots appear posterolaterally on the abdomen. All eyes elevated on cephalothorax and approximately equal in size, anterior median eyes 0.08 in diameter, anterior lateral eyes 0.12 in diameter. All eyes located within one eye diameter from each other, except the posterior medians, which are 0.13 apart. Leg I femur 2.23, patella 0.65, tibia 1.63, metatarsus 2.24, tarsus 0.61. All legs pale yellow, with the metatarsus of legs I, II, and IV slightly browner.

Epigynum with very large and robustly sclerotized spermathecae and relatively small copulatory openings, with clearly sclerotized edges (Fig. 2G–I).

**Male**: Total length 3.52. Cephalothorax 1.10 long, 0.88 wide, 0.64 high, yellow with slightly darker shading laterally. Sternum 0.75 long, 0.51 wide, extending half way between coxae IV, light yellow. Abdomen 2.45 long, 0.89 wide, 0.88 high. All eyes elevated on cephalothorax and approximately equal in size, anterior median eyes 0.08 in diameter, anterior lateral eyes 0.13 in diameter. All eyes located within one eye diameter apart from each other, except the posterior medians, which are 0.14 apart. Leg I femur 2.19, patella 0.51, tibia 1.53, metatarsus 2.28, tarsus 0.55. All legs yellow.

Male pedipalp with all sclerites tightly packed at the distal end of the tegulum, leaving the tegulum largely exposed, embolus relatively short (Fig. 2J).

#### Etymology.

This species epithet refers to the municipality of Santo Antônio de Leverger, the location in which the holotype female was collected.

## Discussion

The recent phylogenetic analysis of *Spintharus* (Dziki et al. 2015) and subsequent naming of 15 new species (Agnarsson et al. 2018) has shown a previously underestimated diversity in the genus. The varying morphology of specimens found in Brazil alone suggests there remain a number of additional species to be found in the New World. Among the specimens examined, we found a number of putative species. However, absent DNA data was shown to be critical for diagnoses in this genus by Dziki et al. (2015), and with color patterns – also putatively informative for taxonomy – eroded by ethanol, we opted to highlight this newly discovered diversity by redescribing the only known species from Brazil, *S. gracilis*, and naming a single new species. We chose to describe this species because it is clearly morphologically distinct, and with sufficient material available of both sexes for an accurate description.

Given geographical variation observed in the samples here, and the clear patterns from the Caribbean of short-range endemics, we expect additional sampling of fresh DNA grade material throughout South America to lead to the discovery of very numerous new species. Thus, South American *Spintharus* could emerge as a radiation of charismatic mesofauna with obvious conservation implications and a great potential tool to further understanding of biogeographical patterns in the Americas. *Spintharus* also holds promise as a potentially rich study system in the evolution of color patterns and polymorphism.

## Supplementary Material

XML Treatment for Spintharus gracilis

XML Treatment for Spintharus leverger
